# Influence of Membrane Salt Rejection Properties on Cake-Enhanced Concentration Polarization Effects During Colloidal Fouling of Nanofiltration Membranes

**DOI:** 10.3390/membranes15070215

**Published:** 2025-07-19

**Authors:** Oranso Themba Mahlangu, Bhekie Brilliance Mamba

**Affiliations:** Institute for Nanotechnology and Water Sustainability, College of Science, Engineering and Technology, University of South Africa, Florida Science Campus, Roodepoort 1709, South Africa

**Keywords:** cake-enhanced concentration polarization, solution-diffusion model, membrane fouling, nanofiltration, colloidal fouling, combined fouling

## Abstract

The build-up of a fouling layer on the membrane surface is believed to deteriorate flux and salt rejection by hindering back-diffusion of rejected salts, a phenomenon called cake-enhanced concentration polarization (CECP). Nevertheless, CECP effects have not been linked to the salt rejection properties of the membrane. Furthermore, the decline in salt rejection during fouling has not been related to the decreasing flux, to elucidate the effects of flux on solution rejection as described by the solution-diffusion (SD) model. Therefore, this work examined whether CECP is substantial in membranes with poor salt-rejection properties. Fouling was performed using sodium alginate, Al_2_O_3_, latex, and SiO_2_. The effects of fouling on salt rejection were studied using two nanofiltration (NF) membranes, namely NF270 membrane (46% NaCl rejection) and NF90 membrane (>97% NaCl rejection). The measured flux and salt rejection profiles were compared to those predicted by the CECP and SD models. Overall, the flux declined more (30–60%) for the NF90 membrane (contact angle: 50 ± 3°) compared to the NF270 membrane (10–55%, contact angle: 39 ± 2°) under similar hydrodynamic conditions. Moreover, fouling had more effects on NaCl rejection for the NF90 membrane (2–45% decline) compared to the NF270 membrane (10–30% decline). The decrease in NaCl rejection for the NF90 membrane was ascribed to CECP effects and declining flux. Contrary, CECP effects were less important for the NF270 membrane, and rejection declined due to reduction in flux as predicted by the SD model, indicating that CECP may not be predominant in membranes that poorly reject salts.

## 1. Introduction

The rise in water scarcity and pollution has accelerated the application of high-retention membrane processes such as nanofiltration (NF) and reverse osmosis (RO) to augment the supply of high-quality produce [1]. Nanofiltration is considered more reliable and affordable for the treatment of various water sources, including brackish water, seawater, as well as secondary treated wastewater effluent [2]. This is because NF can selectively separate solutes with low molecular weight (200–2000 g/mol) [3]. However, there are still challenges associated with the technology, thus hindering its application and efficacy in producing high-quality permeate from unconventional feed streams. One of the issues with NF application is fouling, which diminishes both permeate flux and quality by reducing solute rejection [2,4,5]. During filtration, the NF membrane can be fouled by several foulants, including organics, inorganic salts, colloids, and microbes [6].

Fouling is a concern, especially when NF is targeted at removing micropollutants (e.g., pharmaceuticals) and salts. According to many publications, fouling diminishes pollutant rejection through a phenomenon called cake-enhanced concentration polarization (CECP) [7,8,9,10]. Concentration polarization (CP) refers to the build-up of rejected solutes on the surface of the high-retention membranes. In the absence of a fouling layer, there is back-diffusion of the rejected solutes to the bulk feed [11]. Fouling hinders back-diffusion of the solutes, resulting in their build-up and subsequent diffusion to the permeate side, hence the term CECP. Cake-enhanced concentration polarization increases the concentration gradient across the membrane interface (i.e., between the permeate side and feed side). In the case of salts, CECP elevates the osmotic pressure gradient across the membrane, leading to a reduction in the driving force for permeation and therefore a decrease in flux. The evidence of CP effects in solute rejection and flux has been reported for various membrane processes [12,13,14], and the effects were more pronounced at high transmembrane flux, indicating the relationship between flux and CP effects. Concentration polarization increases with a rise in permeate flux as well as applied pressure [11,15]. Besides the effects on salt rejection, the role of CECP effects on the removal of micropollutants such as per- and polyfluoroalkyl substances has been reported [16]. In a more recent study, microplastics have been reported to deteriorate the rejection of certain dissolved organic matter by worsening CECP, but enhance the rejection of inorganic salts by enhancing their back-diffusion through the cake layer [5].

It is evident from the literature reports that CECP diminishes both permeate flux and solute rejection [12,13,14]. The dependency of permeate flux on applied pressure has been demonstrated, where the former increases with a rise in the latter [17]. Moreover, solute rejection increases with flux according to the solution-diffusion (SD) model, and this is ascribed to the dilution effects by the solvent [18] (i.e., water in the case of water treatment). The SD model is extensively used to describe water and salt transport in NF and RO processes [19]. During fouling, the permeate flux decreases due to additional resistance to water flow exerted by the fouling layer. From the relationship between flux and rejection, it would be expected that a decrease in flux (due to membrane fouling) would result in a decline in solute rejection even without the presence of substantial CECP effects. However, the role of diminishing flux (due to fouling) on solute rejection is often disregarded, and the decrease in solute rejection is ascribed to CECP effects without validating its presence. Therefore, CECP could be wrongly used as a scapegoat to explain the inefficiency of high-retention NF and RO membranes in removing solutes during fouling. Furthermore, the role of both foulant properties as well as membrane salt-rejection properties on CECP effects are often overlooked. Comprehension of the fouling mechanisms and factors influencing solute rejection during fouling is critical as this may shed light towards the development and optimization of high-retention membranes.

Therefore, the aim of this work was to investigate whether CECP effects are linked to the salt rejection properties of the membranes or not. Additionally, the work sought to investigate whether the presence of CECP effects depends on the foulant type or if all foulants result in CECP effects. Two NF membranes representing low-salt-rejecting membranes (NF270) and high-salt-rejecting membranes (NF90) were selected. The membranes were fouled using sodium alginate, representing organic foulants, as well as Al_2_O_3_, latex, and SiO_2_, which were used as model colloidal foulants. Colloids are generally larger than the membrane pore size; thus, colloidal fouling would not be expected to result in high resistance to water permeation. However, concentration polarization of the colloids increases the osmotic pressure and affects the permeability of membranes [11]. Fouling was also performed with the combination of the organic foulant with colloidal foulants. During fouling, the rejection of salts (NaCl) and flux were monitored at pre-selected intervals. The SD model, as well as the CECP model, were developed based on our previous work [20] and used to explain NaCl rejection (or determine if CECP effects were substantial) during fouling. Findings of this work shed more light on the role of membrane salt rejection properties, as well as foulant type and/or characteristics, in CECP. The results may help prevent using CECP as a victim for the poor solute rejection during fouling of NF membranes, thus enhancing more accurate reporting of the effects of fouling on the membrane performance.

## 2. Materials and Methods

### 2.1. Materials

The NF270 and NF90 membranes were supplied by Dow Filmtec, Minneapolis, Minnesota, United States. Four model foulants, namely sodium alginate (Sigma Aldrich, Johannesburg, South Africa), polystyrene carboxylated latex (EOC, Oudenaarde, Belgium), aluminium oxide or Al_2_O_3_ (Evonik Degussa GmbH, Hanau-Wolfgang, Germany), and silica colloids or SiO_2_ (Nissan Chemicals, Houston, TX, USA), were selected to represent organic and colloidal foulants. Sodium chloride (ACS reagent, ≥99%), magnesium sulphate (ACS reagent, ≥99%), sodium metabisulphite (SMBS), and potassium chloride (ACS reagent, ≥99%) were purchased from Sigma Aldrich, Johannesburg, South Africa.

### 2.2. Membrane and Foulant Characterization

The foulants were characterized for particle size based on dynamic light scattering (DLS) techniques using a photon correlation spectroscope (PCS 100M, Zetasizer 2C, Malvern Instruments, England, UK). Zeta potential measurements were based on electrophoretic mobility and were performed utilizing a Zetasizer 2C (Malvern Instruments, England, UK). Particle size and zeta potential measurements were performed at neutral pH and 10 mM KCl background electrolyte. The hydrophilicity of the membranes was determined through pure water contact angle measurements using a goniometer (DSA 10-MK2, Kruss, Hamburg, Germany). The analyses were based on the sessile-drop approach. Zeta potential measurements of the membranes were performed using a SurPASS Electrokinetic Analyser (Anton Paar GmbH, Graz, Austria) at neutral pH and 10 mM KCl background electrolyte. The measurements were performed in tangential mode, with an applied pressure of 200 mbar and a gap height of 105 μm.

### 2.3. Fouling and Salt Rejection Experiments

**Figure 1:**
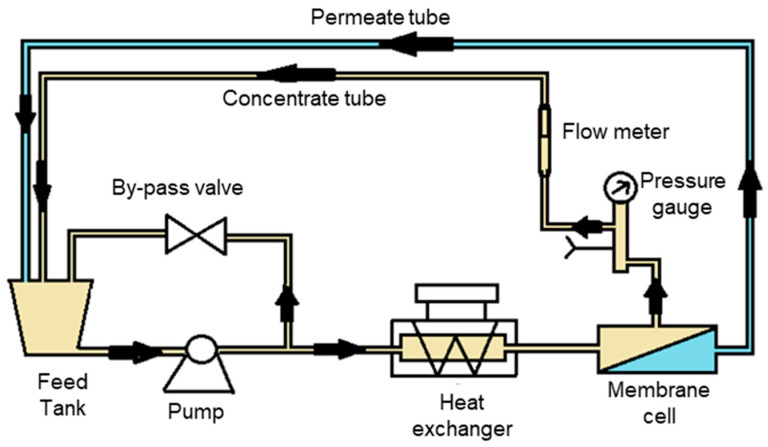
Schematic representation of the cross-flow filtration setup.

All filtration experiments were performed using a customized cross-flow filtration setup (Figure 1). The system was equipped with a piston pump (Rannie High Pressure Pump, No. 82172, 04-03, Ernest Fleming Machinery and Equipment Pty Ltd., Lane Cove West, Australia), a 20 L stainless steel feed tank, and a heat exchanger coupled to a cooling system (Thermo Scientific Neslab RTE–211, Thermo Scientific, Waltham, MA, USA). The membrane cell had a channel length of 2.53 × 10^−1^ m, a channel width of 5.4 × 10^−2^ m, and a height of 1.0 × 10^−3^ m, and thus allowed for experiments to be conducted under laminar cross-flow hydrodynamic conditions. The effective membrane area was 1.37 × 10^−2^ m^2^, and flat sheet membranes were used for all experiments.

Prior to the fouling experiments, the NF270 and NF90 membranes were flushed with tap water and rinsed with DI water to remove sodium metabisulfite (SMBS) solution (0.5 wt.%), which was used as a preservative to reduce biofouling during storage. Thereafter, the membranes were individually compacted with deionized (DI) water at 10 bar for 3 h to obtain stable fluxes. Subsequently, pure water flux measurements were performed at a pressure range of 4–10 bar, and the permeate flux was estimated from Equation (1).

(1) $$J_{w}=\frac{V}{A∆t}$$

where $J_{w}$ is permeate flux (L/m^2^h), V is permeate volume (L), A is effective membrane area (m^2^) and Δt is time taken to collect a specific volume of permeate (h). Afterwards, the membrane’s pure water permeability ($L_{p}$) was calculated from the measured flux and applied pressure (∆P) using Equation (2).

(2) $$L_{p}=\frac{J_{w}}{∆P}$$

Following flux measurement, observed salt rejection properties ($R_{o}$) of the membranes was determined at an applied pressure of 6 bar by individually filtering 10 mM NaCl and 2000 mg/L MgSO_4_ solutions, and rejection was estimated from Equation (3).

(3) $$R_{o}=(1-\frac{C_{p}}{C_{f}})\times 100$$

where $C_{p}$ and $C_{f}$ are solute concentrations in the permeate and feed, respectively. The salt content in the feed and permeate was measured as electrical conductivity using a Consort C6010 electrical conductivity meter, Consort, Turnhout, Belgium.

Subsequent to the determination of salt rejection properties, the feed solution was replaced with the desired foulant solution (Table 1), and the fouling experiments were performed at 6 bar for 12 h. All experiments were performed at 23 ± 2 °C, 15% recovery, 20 L feed volume, and in recycling mode, where both the retentate and permeate were recirculated into the feed tank. The individual foulant concentrations were kept at 30 mg/L for sodium alginate and 50 mg/L each for the colloids (latex, aluminium oxide, and silica). The background electrolyte was 10 mM NaCl for all the fouling experiments, and the cross-flow velocity was maintained at 0.2 m/s to ensure that the variation in concentration polarization effects was not due to the differences in cross-flow velocity [21]. During the fouling experiments, the membrane flux and NaCl rejection were monitored at selected time intervals. At the end of each filtration run, the membrane was removed from the membrane cell and characterized for morphology using a scanning electron microscope (SEM, Jeol JSM IT300, Tokyo, Japan). Before the next filtration run using a different foulant solution, the filtration unit was cleaned by recirculating 0.1% NaOCl for 1 h, followed by rinsing several times with DI water. Thereafter, the filtration system was again recirculated with 0.1 M HCl for 1 h and rinsed with DI water. All experiments were performed in triplicate to determine the reproducibility of the experiments.

### 2.4. Modeling Fouling Effects on NaCl Rejection

#### 2.4.1. Development of the Solution-Diffusion (SD) Model

A solution-diffusion (SD) model that related solute rejection to the water flux was developed and described in our previous work [20]. Briefly, according to the model, the water flux and the solute flux are given by Equation (4) and Equation (5), respectively.

(4) $$J_{w}=\frac{D_{w}\emptyset_{w}C_{w,f}}{∆X}.\left(\frac{V_{w}.\left[∆P-∆\pi \right]}{R.T}\right)=L_{p}[∆P-∆\pi ]$$

(5) $$J_{s}=\frac{D_{s}\emptyset_{s}}{∆X}.[C_{s.f}-C_{s.p}]=B[C_{s.f}-C_{s.p}]$$

where $J_{w}$ is water flux, $J_{s}$ is solute flux, D is the diffusion coefficient, Ø is the partitioning coefficient, C is the concentration, $V_{w}$ is the molar volume, ∆π is the osmotic pressure, R is the gas constant, T is the absolute temperature, $L_{p}$ is the water permeability constant, ∆X is the thickness of the membrane active layer, and B is the solute permeability constant. The subscripts w, s, f, and p represent water, solute, feed, and permeate, respectively. According to Equation (4), the water flux is expected to increase with an increase in applied pressure.

Therefore, the observed rejection ($R_{o}$) can be linked to $J_{w}$ and B via Equation (6). It must be considered that the SD model is only valid if concentration polarization is disregarded.

(6) $$R_{o}=1-\frac{C_{s.p}}{C_{s.f}}=\frac{J_{w}}{{(J}_{w}+B)}=\frac{L_{p}[∆P-∆\pi ]}{\left[L_{p}\left(∆P-∆\pi \right)+B\right]}$$

It is clear from Equation (6) that rejection increases with an increase in permeate flux due to dilution effects [22]. The relationship between permeate flux and salt rejection was verified by carrying out additional experiments using the clean membrane without the addition of foulants. The applied pressure (∆P) was increased to the desired value, and the solute permeability constant (B) was estimated from the calculated rejection and corresponding flux data based on Equation (6).

#### 2.4.2. Development of the Cake-Enhanced Concentration Polarization (CECP) Model

The cake-enhanced concentration polarization (CECP) model has been used in previous studies [23,24] including our earlier work [20]. The concentration polarization (CP) factor (β) is defined by the film theory (Equation (7)), which accounts for the solute concentration on the membrane surface ($C_{m}$), permeate ($C_{p}$) and in the feed ($C_{f}$) [25].

(7) $$\beta =\frac{C_{m}-C_{p}}{C_{f}-C_{p}}=exp(\frac{J_{w}}{k})$$

The CP factor is also linked to the water flux and solute mass transfer coefficient (k), wherein the mass transfer coefficient can be determined from Equations (8) and (9) for a laminar flow in a thin rectangular channel [23,26] (like in the case of the current work).

(8) $$k=0.808[{\frac{{6QD}^{2}}{{WH}^{2}L}]}^{\frac{1}{3}}\;$$

(9) $$S_{h}=\frac{{kd}_{h}}{D_{\infty }}=0.065{Re}^{0.875}{S_{c}}^{0.25}$$

where Q is the feed flow rate, D is the solution diffusion coefficient, W is the channel width, H is the channel height, L is the channel length, $S_{h}$ is the Sherwood number, $d_{h}$ is the hydrodynamic diameter, Re is the Reynolds number and $S_{c}$ is the Schmidt number. The CP factor reduces the permeate flux ($J_{w}$) by decreasing the driving force (∆P) through Equation (10).

(10) $$J_{w}=\frac{∆P-\beta ∆\pi }{\mu R_{m}}$$

where μ is electrolyte viscosity and $R_{m}$ is the transmembrane resistance. Fouling prevents back-diffusion of solutes from the membrane surface to the feed. Subsequently, this increases solute concentration on the membrane surface ($C_{m}$) in a phenomenon called cake-enhanced concentration polarization (CECP). To differentiate between CP for the clean membrane and that of the fouled membrane, the former is presented as β while the latter is denoted $\beta_{CECP}$. Subsequently, the hindered mass transfer coefficient (k*) is related to the hindered diffusion coefficient via Equation (11), which accounts for the cake thickness ($\delta_{c})$ and the hindered diffusion coefficient (D*). The hindered diffusion coefficient is defined as $D*=D\varepsilon_{c}\tau^{-1}$ where $\varepsilon_{c}$ is cake porosity and τ is tortuosity = $1-ln(\varepsilon_{c}^{2})$ [27].

The flux equation was therefore modified to incorporate CECP effects as well as cake resistance ($R_{c}$) according to Equation (11), where the hindered mass transfer coefficient (k*) is included in the equation as it links to the osmotic pressure drop through: $∆\pi =f_{os}\left(C_{m}-C_{p}\right)=f_{os}C_{b}R_{o}exp\frac{J_{w}}{k*}$, where $f_{os}$ depicts an osmotic coefficient used to convert the molar salt concentration to osmotic pressure. Since low NaCl concentrations were used in this work, the Van’t Hoff equation was used where $f_{os}=2RT$ (R is the gas constant and T is absolute temperature) [24].

(11) $$J_{w}=\frac{∆P-\beta_{CECP}∆\pi }{\mu (R_{m}+R_{c})}$$

The cake resistance ($R_{c}$) can be estimated from the deposited cake layer thickness ($\varepsilon_{c}$), porosity, particle density ($\rho_{p}$) and particle diameter ($d_{p}$) based on the Carman–Kozeny relationship (Equation (12)) [28].

(12) $$R_{c}=\left[\frac{180\left(1-\varepsilon_{c}\right)}{\rho_{p}d_{p}^{2}\varepsilon_{c}^{3}}\right]\varepsilon_{c}[\rho_{p}\left(1-\varepsilon_{c}\right)]$$

In this work, the porosity was estimated to be 0.4 and the cake thickness ($\varepsilon_{c}$) was adjusted to fit the CECP flux model (Equation (11)) to the flux measured during the fouling experiments. Subsequently, the observed rejection ($R_{o}$) (Equation (13)) was linked to the CECP factor ($\beta_{CECP}$) and intrinsic rejection ($R_{i}$), by assuming a constant $R_{i}=1-C_{p}/C_{m}$ [29].

(13) $$R_{o}=1-\frac{1-R_{i}}{\left[1-R_{i}+(\frac{R_{i}}{\beta_{CECP}})\right]}$$

## 3. Results and Discussion

### 3.1. Foulant and Membrane Properties

Table 2 shows the particle sizes and zeta potentials of the model foulants at neutral pH and 10 mM KCl background electrolyte. The foulants were characterized by particle size <170 nm (122 nm–161 nm) and were negatively charged at neutral pH (except for Al_2_O_3_). The foulants were expected to foul the membranes differently due to their contrasts in physicochemical properties.

According to the literature, the NF270 membrane has a skin layer consisting of polyamide, and the membrane has the following characteristics: average pore radius of 0.43 nm, molecular weight cut-off of 200–300 Da, MgSO_4_ rejection of 97%, NaCl rejection of 50%, contact angle of 54.8°, and isoelectric point at pH 4 [17]. On the other hand, the active layer of the NF90 membrane comprises semi-aromatic piperazine-based polyamide supported on layers of polysulfone and polyester [30]. The manufacturer indicated that the NF90 membrane has >97% NaCl and MgSO_4_ rejection. Our analysis revealed that the NF270 and NF90 membranes were hydrophilic and the NF270 membrane was the most hydrophilic with a water contact angle of 39 ± 2° compared to 50 ± 3° for the NF90 membrane (Table 3). Subsequently, the NF270 had a higher pure water permeability compared to the NF90 membrane, and this could be ascribed to the lower membrane resistance of the NF270 membrane. However, the NF90 membrane achieved higher salt rejection compared to the NF270 membrane, which had < 50% NaCl and 96 ± 3% MgSO_4_ rejection. Both the membranes were negatively charged at neutral pH and had similar zeta potentials. According to the literature, the NF270 membrane is relatively smooth with a surface roughness of 8.6 nm [10], while the NF90 membrane is rough with a mean surface roughness of 77 nm [31]. Therefore, the NF90 membrane was expected to foul more than the NF270 membrane under similar operational conditions.

### 3.2. Membrane Fouling Propensity

**Figure 2:**
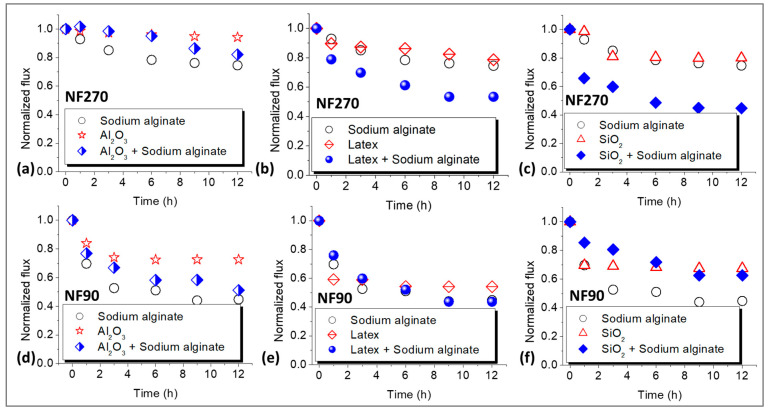
Normalized flux decline profiles for the NF270 (**a**–**c**) and NF90 (**d**–**f**) membranes due to organic, colloidal, and combined fouling by Sodium alginate (30 mg/L), Al_2_O_3_ (50 mg/L), Latex (50 mg/L), and SiO_2_ (50 mg/L). The normalized flux (unitless) was calculated by dividing the flux measured during the fouling experiments by the initial flux at the beginning of the fouling experiments. All experiments were performed at applied pressure of 6 bar, 0.2 m/s cross flow velocity, 10 mM NaCl background electrolyte, and temperature of 23 ± 2 °C.

Figure 2 shows the flux decline profiles for the NF270 and NF90 membranes. Sodium alginate was used to compare organic fouling to colloidal fouling, as well as the effects of combined fouling on permeate flux. Mainly, there was more fouling by sodium alginate for the NF90 membrane (60%, Figure 2d) compared to the NF270 membrane (28%, Figure 2a) at the end of the 12 h filtration run. Fouling by Al_2_O_3_ resulted in negligible flux decline for the NF270 membrane (Figure 2a), while the effect was substantial for the NF90 membrane (Figure 2d). Combined fouling by Al_2_O_3_ + sodium alginate decreased flux for both membranes and the effect was more severe for the NF90 membrane (50%) compared to the NF270 membrane (20%). However, no synergistic effects were observed for both membranes when fouling was performed with combined Al_2_O_3_ + sodium alginate. Generally, fouling by latex resulted in more flux decline (50%) for the NF90 membrane (Figure 2e) compared to the NF270 membrane (Figure 2b), which suffered about 20% flux decline after 12 h of filtration. Combined latex + sodium alginate fouling promoted flux decline leading to 50% flux decline for the NF270 membrane and 55% flux decline for the NF90 membrane. The flux profile of the NF90 membrane for fouling with latex + sodium alginate mirrored that of sodium alginate, possibly showing more influence of alginate on fouling. Again, more fouling was observed for the NF90 membrane compared to its NF270 membrane counterpart. Moreover, there was more flux decline for the NF90 membrane (Figure 2f) when fouling was performed with SiO_2_ (30%) compared to fouling of the NF270 membrane (Figure 2c) with the same foulant (20%). Combined SiO_2_ + sodium alginate aggravated fouling for the NF270 membrane, however, for the NF90 membrane, the flux decline was slightly higher than that of SiO_2_. Nonetheless, fouling was still lower than that of individual fouling by sodium alginate indicating the absence of synergistic effects.

Basically, there was more fouling of the NF90 membrane compared to the NF270 membrane. This could be ascribed to the NF90 membrane being rougher (mean surface roughness of 77 nm [31]) and less hydrophilic (water contact angle of 50 ± 3°, Table 2). These properties promoted attachment of foulants on the membrane surface. Fouling is believed to reduce flux due to the introduction of additional hydraulic resistance to water flow by the cake layer and blocking of the membrane microvoids. However, there are also reports of fouling reducing flux by increasing the osmotic pressure difference across the membrane interface through CECP effects [2]. No synergistic effects were observed for combined fouling with Al_2_O_3_ + sodium alginate for both the NF270 and NF90 membranes. This could indicate potential disruption of the formation of a uniform fouling layer on the membrane surface when Al_2_O_3_ is present. Furthermore, fouling of the NF270 membrane by Al_2_O_3_ resulted in negligible flux decline, thus, no synergy was expected when Al_2_O_3_ was combined with sodium alginate. For combined latex + sodium alginate, there was evidence of synergistic effects for both the NF270 and NF90 membranes indicating that the formation of dense cake layers was not disrupted. Synergistic effects existed for combined fouling of the NF270 membrane with SiO_2_ + sodium alginate but were non-existent for the NF90 membrane fouled under similar conditions. These inconsistencies in the fouling trends need further investigation to explain the differences in the fouling patterns.

### 3.3. Effect of Fouling on Salt (NaCl) Rejection

**Figure 3:**
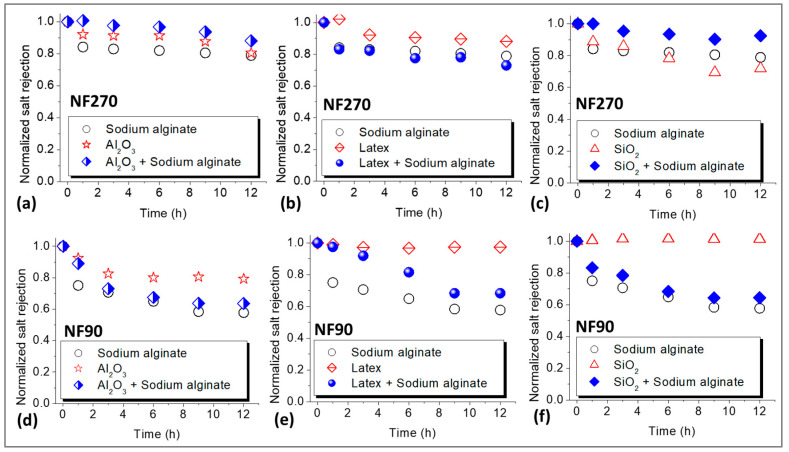
Normalized NaCl rejection profiles for the NF270 (**a**–**c**) and NF90 (**d**–**f**) membranes due to organic, colloidal, and combined fouling by Sodium alginate (30 mg/L), Al_2_O_3_ (50 mg/L), Latex (50 mg/L), and SiO_2_ (50 mg/L). The normalized rejection (unitless) was calculated by dividing NaCl rejection measured during the fouling experiments by the initial NaCl rejection at the beginning of the fouling experiments. All experiments were performed at applied pressure of 6 bar, 0.2 m/s cross flow velocity, 10 mM NaCl background electrolyte, and temperature of 23 ± 2 °C.

The effects of fouling on NaCl rejection were investigated and the results are presented in Figure 3. Fouling by sodium alginate resulted in a decrease in NaCl rejection for both the NF270 membrane (Figure 3a) and the NF90 membrane (Figure 3d), and the decline in rejection was more severe for the latter membrane (45%) compared to the former membrane (20%). Colloidal fouling of the NF270 membrane resulted in 20%, 12%, and 30% reduction in the rejection of NaCl for fouling with Al_2_O_3_, latex and SiO_2_, respectively (Figure 3a–c). Combined fouling only promoted the decline in NaCl rejection for fouling with latex + sodium alginate, while Al_2_O_3_ + sodium alginate as well as SiO_2_ + sodium alginate resulted in lesser decline in NaCl rejection compared to individual fouling by the colloids.

Fouling mainly resulted in more decrease in NaCl rejection for the NF90 membrane (Figure 3d–f) compared to the NF270 membrane. This was exceptional for fouling with latex (about 2%) and SiO_2_ (0%) where NaCl rejection remained relatively unchanged. Unlike the NF270 membrane, combined fouling of the NF90 membrane resulted in substantial decline in NaCl rejection. However, the effect was less severe compared to individual fouling by sodium alginate. In most cases, NaCl rejection profiles for combined fouling mirrored those of individual fouling by sodium alginate. The decrease in salt rejection due to membrane fouling has been reported before and ascribed to CECP effects [30], wherein the increase in salt concentration in the vicinity of the membrane surface resulted in subsequent diffusion of the salts to the permeate side, hence the lower rejection. As alluded before, solute rejection increases with permeate flux. Therefore, the decrease in NaCl rejection could be due to the decline in flux (i.e., dilution effects) as observed in Figure 2. However, many researchers overlook the role of flux in rejection when explaining decrease in solute rejection due to fouling. Nonetheless, there are some studies that have linked solute rejection to permeate flux to gain insights into the effects of fouling on rejection [8,29]. Even so, further investigation is needed to acknowledge the role of flux (during fouling) on salt rejection and how this links to the membrane salt rejection properties. This is interrogated in this work and discussed in the next sections.

### 3.4. Verification of Cake-Enhanced Concentration Polarization (CEC) Effects on Salt Rejection

**Figure 4:**
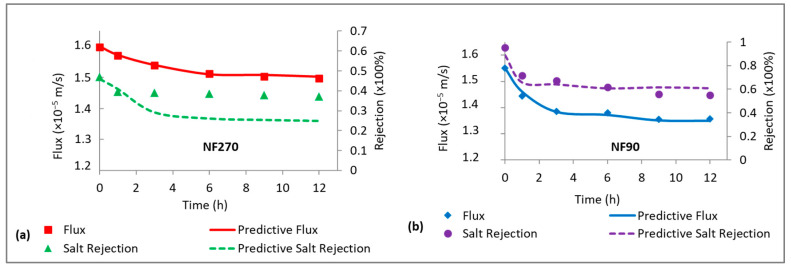
Measured and predictive permeate flux and salt rejection for fouling of the NF270 (**a**) and NF90 (**b**) membranes with 30 mg/L Sodium alginate. All experiments were performed at applied pressure of 6 bar, 0.2 m/s cross flow velocity, 10 mM NaCl background electrolyte, and temperature of 23 ± 2 °C.

The CECP model (Equation (13)) was used to predict the role of cake-enhanced concentration polarization on the rejection of NaCl by the NF270 and NF90 membranes fouled by sodium alginate. There was good prediction of flux by the model for both membranes (Figure 4). However, there was no evidence of CECP for the NF270 membrane as the model underpredicted NaCl rejection (Figure 4a), while the decline in NaCl rejection for the NF90 membrane was due to CECP effects as predicted by the model (Figure 4b). These observations indicate that CECP, to some extent, relates to the salt rejection properties of the membrane. The decline in NaCl rejection for the NF270 membrane was probably due to the declining flux according to Equation (6). On the other hand, NaCl rejection for the NF90 membrane declined due to a combination of both flux and CECP effects. The role of permeate flux in rejection will be verified in a separate section.

### 3.5. Evidence of CECP Effects in Low-Salt-Rejecting Membranes (NF270)

**Figure 5:**
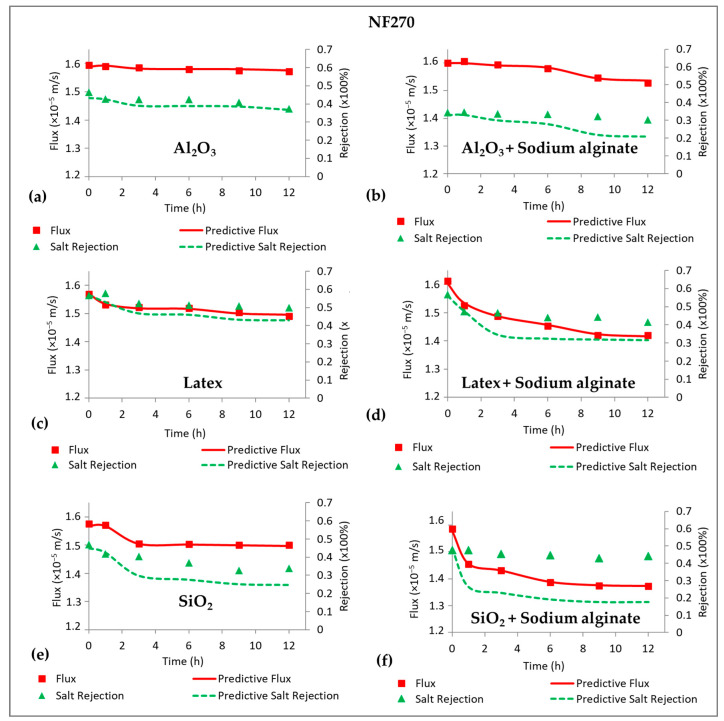
Measured and predictive permeate flux and salt rejection for fouling of the NF270 membrane with Al_2_O_3_ (50 mg/L), Latex (50 mg/L), SiO_2_ (50 mg/L), and their combination with Sodium alginate (30 mg/L). All experiments were performed at applied pressure of 6 bar, 0.2 m/s cross flow velocity, 10 mM NaCl background electrolyte, and temperature of 23 ± 2 °C.

Figure 5 shows predictive NaCl rejection for the NF270 membrane fouled by Al_2_O_3_, latex, SiO_2_ and their combination with sodium alginate. The CECP model failed to predict NaCl rejection for all the fouling types except for membrane fouling by Al_2_O_3_ (Figure 5a). In all cases, the model underpredicted rejection, indicating that the decline in NaCl rejection for the NF270 membrane was not due to CECP effects, but other factors played a role. The decrease in NaCl rejection when the membrane was fouled with Al_2_O_3_ was due to CECP effects. This could be supported by the evidence of negligible decline in permeate flux (Figure 2a), while the rejection of NaCl decreased substantially. As observed in Figure 4a, fouling of the NF270 by sodium alginate did not result in remarkable CECP effects. Similarly, combined fouling for all the colloids did not result in CECP effects. In their work, Wang et al. [1] reported a lower concentration polarization factor the NF270 membrane and concluded that a higher ion permeation rate corresponded to a reduced ion concentration at the membrane surface. Subsequently, this leads to a decrease in concentration polarization and the associated polarization factor. Therefore, cake-enhanced concentration polarization effects are expected to be less substantial for low-salt-rejecting membranes, contrary to high-salt-rejecting membranes, where the highly rejected salts accumulate at the membrane vicinity.

### 3.6. Evidence of CECP Effects in High-Salt-Rejecting Membranes (NF90)

**Figure 6:**
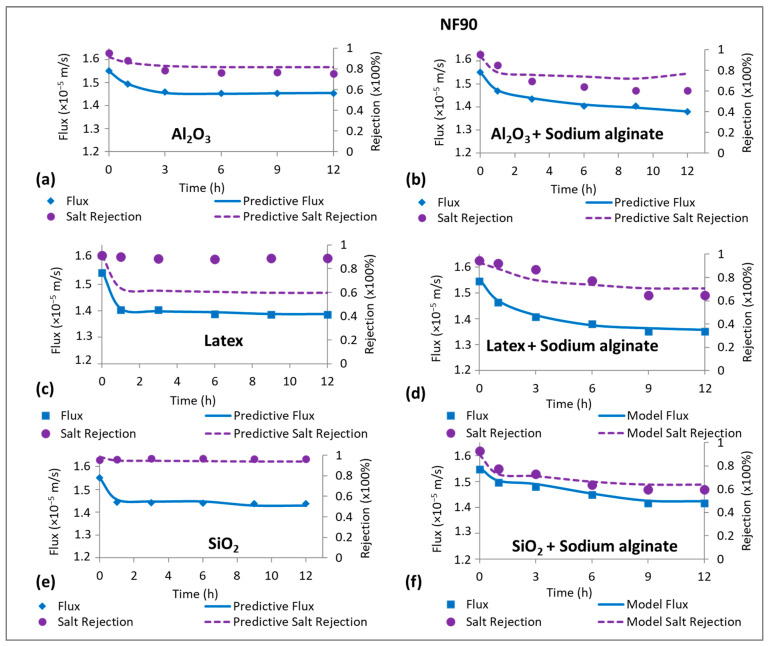
Measured and predictive permeate flux and salt rejection for fouling of the NF90 membrane with Al_2_O_3_ (50 mg/L), Latex (50 mg/L), SiO_2_ (50 mg/L), and their combination with Sodium alginate (30 mg/L). All experiments were performed at applied pressure of 6 bar, 0.2 m/s cross flow velocity, 10 mM NaCl background electrolyte, and temperature of 23 ± 2 °C.

Figure 6 shows the measured and predicted flux and NaCl rejection for the NF90 membrane. The predictive results from the CECP model (Equation (13)) fit NaCl rejection data for all the fouling types except for membrane fouling with latex (Figure 6c). These findings indicated that the decrease in flux as well as decline in NaCl rejection were due to CECP effects for the high-salt-rejecting membrane. However, this does not imply that CECP was the only factor controlling flux decline and decrease in NaCl rejection. The absence of CECP effects for fouling with latex could imply that the reduction in flux could be ascribed to cake layer formation that exerted additional resistance to water flow. Furthermore, fouling by latex resulted in the formation of a more compressible cake layer leading to flux decline, while NaCl rejection remained relatively unchanged. Unlike the low-salt-rejecting membrane (NF270), CECP effects were more substantial for the NF90 membrane, indicating that cake-enhanced concentration polarization effects could be related to the salt rejection properties of the membrane. It was noted in Figure 4b that fouling of the NF90 membrane by sodium alginate resulted in CECP effects. Consequently, all combined fouling experiments resulted in CECP effects and this could be ascribed to the sodium alginate having more effect in preventing the back-diffusion of NaCl from the membrane surface (i.e., greater $\beta_{CECP}$ than the colloidal foulants). This hypothesis could be supported by no evidence of CECP effects for fouling of the NF90 membrane with latex (Figure 6c), but upon fouling with combined latex + sodium alginate, CECP prevailed (Figure 6d). Previous studies also reported CECP effects for colloidal fouling using silica colloids [11] and other foulants such as microalgae [13]. It was recommended that concentration polarization effects could be alleviated by operating at high cross-flow velocity. Flux decline during fouling is presumed to be due to a combination of concentration polarization effects and cake filtration, wherein concentration polarization enhances fouling formation and can contribute to flux decline by over 50%.

The cake-enhanced concentration polarization factors ($\beta_{CECP}$) for the different fouling layers were estimated based on Equation (7) and the results are presented in Table 4. It was noted that when the NF270 and NF90 membranes were fouled by the same foulant, $\beta_{CECP}$ for the NF90 membrane was always greater than that for the NF270 membrane. Additionally, it was observed that the presence of organic foulant (sodium alginate) in combined organic-colloidal fouling of the NF90 membrane resulted in higher $\beta_{CECP}$ compared to that of individual organic and colloidal fouling. There was no apparent evidence of synergistic effects of foulants on $\beta_{CECP}$ for combined fouling of the NF270 membrane (except for combined fouling by Latex + Sodium alginate). This could be ascribed to the negligible $\beta_{CECP}$ effects for the low-salt-rejecting membrane. Contrarily, synergistic effects of foulants on $\beta_{CECP}$ for combined fouling of the NF90 membrane were present. Again, this was because both organic and colloidal foulants resulted in notable cake-enhanced concentration polarization ($\beta_{CECP}$).

### 3.7. Evidence of Declining Flux Effects on Salt Rejection

**Figure 7:**
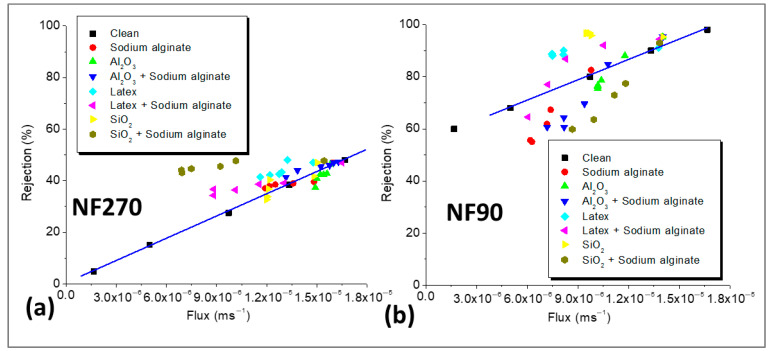
NaCl rejection as a function of permeate flux for the NF270 (**a**) and NF90 (**b**) membranes. All experiments were performed at applied pressure of 6 bar, 0.2 m/s cross flow velocity, 10 mM NaCl background electrolyte, and temperature of 23 ± 2 °C. The foulant concentrations were 30 mg/L sodium alginate, Al_2_O_3_, 50 mg/L latex, 50 mg/L, and 50 mg/L SiO_2_.

The solution-diffusion (SD) model was used to investigate the effects of declining flux on NaCl rejection, and the results are presented in Figure 7. NaCl rejection was plotted as a function of flux for the clean and fouled membranes, and NaCl rejection declined with the decrease in permeate flux, showing the influence of flux on salt rejection. The observed rejections of NaCl by the fouled NF270 membrane (Figure 7a) were mostly higher than those of the clean membrane at similar flux, except for membrane fouling with Al_2_O_3_. Since CECP effects were not substantial for the low-salt-rejecting membrane (as indicated in Figure 5), the observed decrease in rejection over time was due to decrease in flux, leading to the reduction in dilution effects. When the rejection for the fouled membrane was higher than that of the clean membrane (at similar flux), then the fouling layer improved rejection by acting as an additional sieve. This often misleads researchers (who observe an overall decrease in rejection over time) to conclude that the fouling layer decreased rejection because the rejection for the fouled membrane is not compared to that of the clean membrane at the same flux. In this work, NaCl rejection for the clean membrane was compared to that of fouled membrane under the same permeate flux to determine if the fouling layer improved rejection or not. The increased decline in NaCl rejection for fouling with Al_2_O_3_ (compared to the clean membrane under the same flux, Figure 7a) indicated the presence of CECP effects as confirmed in Figure 5a, where Al_2_O_3_ deposition on the membrane surface resulted in negligible flux decline, but the decrease in NaCl rejection was substantial.

The rejection of NaCl for the fouled NF90 membrane decreased with a decline in permeate flux (Figure 7b). Except for fouling with latex and SiO_2_, the decrease in rejection for the other foulants was more than that of the clean membrane at similar flux, showing additional CECP effects on NaCl rejection. When fouling was performed with latex and SiO_2_, the decrease in rejection was lower than that predicted by the SD model, indicating that CECP effects were limited. In this case, the fouling layer restricted salt passage leading to higher NaCl rejection than the clean membrane at similar flux. Nonetheless, an overall decrease in rejection (negligible) was observed due to the reduction in dilution effects (decrease in flux), and this observation can lead to the conclusion that fouling decreased rejection, yet NaCl removal was higher than that of the clean membrane under the same flux. Previous studies have shown that the rejection of salts increases with permeate flux [19,32], and this was in agreement with the findings of this work. Upon fouling, NaCl rejection decreased with flux; however, the presence of CECP worsened the decrease, and this was dependent on the type of foulant.

### 3.8. Membrane Autopsy

**Figure 8:**
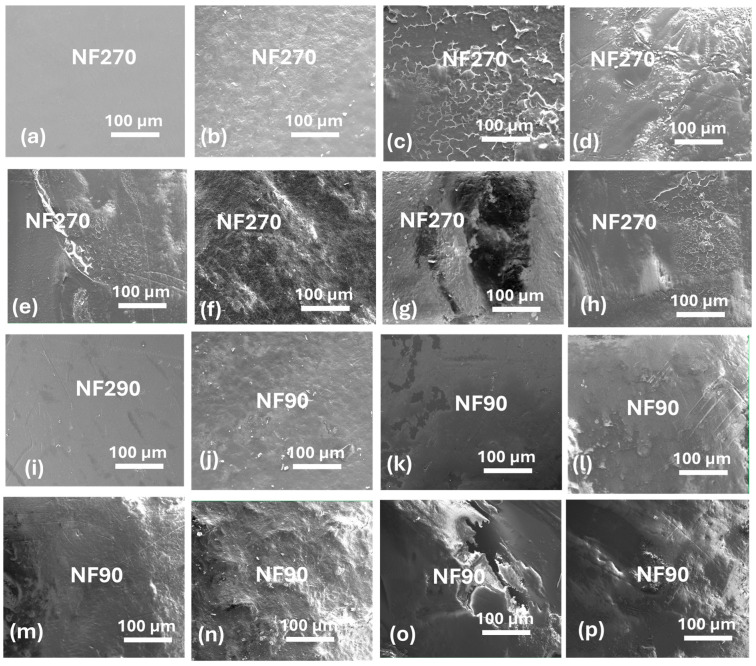
Scanning electron micrographs of clean and fouled NF270 and NF90 membranes: clean membranes (**a**,**i**), fouling by Sodium alginate (**b**,**j**), Al_2_O_3_ (**c**,**k**), Al_2_O_3_ + Sodium alginate (**d**,**l**), Latex (**e**,**m**), Latex + Sodium alginate (**f**,**n**), SiO_2_ (**g**,**o**), and SiO_2_ + Sodium alginate (**h**,**p**). The membranes were fouled with 30 mg/L sodium alginate, 50 mg/L Al_2_O_3_, 50 mg/L, Latex, and 50 mg/L SiO_2_ under applied pressure of 5 bar, 0.2 m/s cross flow velocity, 10 mM NaCl background electrolyte and temperature of 23 ± 2 °C.

Figure 8 shows scanning electron micrographs of the NF270 and NF90 membranes after fouling. Fouling with sodium alginate resulted in the formation of dense fouling layers in both the NF270 (Figure 8b) and NF90 (Figure 8j) membranes. Dense fouling layers were also formed for combined organic + colloidal fouling. However, the fouling layers appeared less compact for combined Al_2_O_3_ + sodium alginate (Figure 8d) and SiO_2_ + sodium alginate (Figure 8h), and this probably explains the lower flux decline observed in Figure 3a,c. Table 4 shows the estimated cake layer thicknesses for the NF270 and NF90 membranes after fouling. It was observed that membrane fouling by sodium alginate resulted in thicker cake layers ($\varepsilon_{c}$) than those of the colloids. Subsequently, the dense fouling layers resulted in higher cake resistance ($R_{c}$). However, $R_{c}$ for the fouling of the NF90 membrane was always larger than that of the NF270 membrane (for the membranes fouled under similar experimental conditions). Similarly, the cake layer thickness ($\varepsilon_{c}$) for NF90 membranes were generally larger than that of the NF270 membranes. Nonetheless, it was observed that fouling was promoted when both the organics and colloids co-existed in the feed, resulting in thicker fouling layers, which corresponded with higher cake resistance to hydraulic flow. These findings demonstrated that various factors contribute to flux decline during membrane fouling.

The presence of colloids (Al_2_O_3_ and SiO_2_) appeared to disturb the formation of uniform organic fouling layers on the surface of the NF270 membrane (Figure 8c,g). However, the formation of dense cake layers was not disturbed by the presence of Al_2_O_3_ (Figure 8j) and SiO_2_ (Figure 8n) for combined fouling of the NF90 membrane. These inconsistencies indicate that the fouling patterns may also be influenced by membrane salt rejection properties.

It was believed that the presence of Al_2_O_3_ and SiO_2_ in combined fouling for the NF270 membrane prevented the formation of a uniform fouling layer, resulting in no evidence of synergistic effects. Surface micrographs of the fouled membranes showed that the fouling layer for Al_2_O_3_ + sodium alginate was not uniform and/or dense (Figure 8d) compared to that of sodium alginate alone (Figure 8b). This probably explained the lower flux decline (18%) compared to fouling by only sodium alginate (26%) observed in Figure 2a. Similarly, the fouling layer micrograph of the NF270 membrane fouled by SiO_2_ + sodium alginate appeared loose and less compact (Figure 8h). All the fouling layer micrographs for the combined fouling of the NF90 membrane appeared dense and compact (Figure 8l,n,p), and this explains the more flux decline noted in Figure 2d–f. Although the cake layers for combined fouling of the NF90 membrane appeared dense and compact, the resultant flux decline profiles (except for Latex + Sodium alginate) were slightly lower than that of fouling by alginate alone again demonstrating possible formation of more porous cake layers with reduced or no synergistic CECP effects.

## 4. Conclusions

The solution-diffusion (SD) and the cake-enhanced concentration polarization (CECP) models were used to investigate if the concentration polarization (CP) effects prevail in membranes with poor salt rejection properties, and if all foulants result in CECP effects or not. Two nanofiltration (NF) membranes, representing low-salt-rejecting membranes (NF270) and high-salt-rejecting membranes (NF90), were selected. It was found that fouling was more for the NF90 membrane (30–60%) compared to the NF270 membrane (10–55%) and this was ascribed to the lower hydrophilicity and rougher surface of the NF90 membrane. Mostly, fouling decreased the rejection of NaCl over time for both membranes. However, the decrease was more pronounced for the NF90 membrane in most instances (2–45%) and depended on the foulant type. The CECP model revealed that organic fouling by sodium alginate did not result in notable CECP effects for the NF270 membrane, while CECP effects were substantial for the NF90 membrane due to its high-salt rejection properties. Colloidal fouling and combined organic + colloidal fouling did not result in remarkable CECP effects for the NF270 membrane (except for fouling with Al2O_3_). On the other hand, CECP effects were obvious for the NF90 membrane (except for fouling with latex). The SD model revealed that the decline in NaCl rejection for the NF270 membrane was due to a reduction in permeate flux, and the rejection was mostly higher than that of the clean membrane at similar flux. Contrary, the decline in NaCl reduction for the NF90 membrane was more severe than that of the clean membrane at similar flux, indicating drastic CECP effects. Findings of this work indicate that CECP is not the only factor leading to the decline in solute rejection for high-retention membranes, but also the decrease in flux. Furthermore, CECP depends on the solute rejection properties of the membrane, together with the type of foulant and its characteristics.

## Figures and Tables

**Table 1:** Properties of the fouling solutions.

Foulant Solution	Foulant (mg/L)				NaCl (mM)
	Sodium Alginate	Latex	Al_2_O_3_	SiO_2_	
Sodium alginate	30	0	0	0	10
Latex	0	50	0	0	10
Latex + Sodium alginate	30	50	0	0	10
Al_2_O_3_	0	0	50	0	10
Al_2_O_3_ + Sodium alginate	30	0	50	0	10
SiO_2_	0	0	0	50	10
SiO_2_ + Sodium alginate	30	0	0	50	10

**Table 2:** Particle size and zeta potential of the model foulants.

Model Foulant	Particle Size (nm)	Zeta Potential (mV)
Sodium alginate	122.3 ± 5.2	−34.2 ± 2.3
Latex	158.5 ± 5.2	−41.8 ± 3.2
Al_2_O_3_	161.5 ± 2.8	38.2 ± 1.5
SiO_2_	135.4 ± 4.2	−28.8 ± 2.1

**Table 3:** Characteristics of the NF270 and NF90 membranes.

Parameter	Membrane	
	NF270	NF90
Contact angle (°)	39 ± 2	50 ± 3
Pure water permeability (Lm^−2^ h^−1^ bar^−1^)	13 ± 2	7.1 ± 3
MgSO_4_ rejection (%)	96 ± 3	98 ± 2
NaCl rejection (%)	45 ± 4	96 ± 3
Zeta potential (mV)	−15 ± 3.2	−18 ± 1.4
Membrane resistance (m^−1^)	3.8 ± 1.1 × 10^13^	4.1 ± 0.8 × 10^13^

**Table 4:** Cake-enhanced concentration polarization factors ($\beta_{CECP}$), cake thickness ($\varepsilon_{c}$), and cake resistance ($R_{c}$) during organic, colloidal, and combined fouling of NF270 and NF90 membranes. All experiments were performed at applied pressure of 6 bar, 0.2 m/s cross flow velocity, 10 mM NaCl background electrolyte, and temperature of 23 ± 2 °C. The foulant concentrations were 30 mg/L sodium alginate, Al_2_O_3_, 50 mg/L latex, 50 mg/L, and 50 mg/L SiO_2_.

	NF270			NF90		
	$\beta_{CECP}$	$\varepsilon_{c}$ (µm)	$R_{c}$ (×10^12^ 1/m)	$\beta_{CECP}$	$\varepsilon_{c}$ (µm)	$R_{c}$ (×10^12^ 1/m)
Sodium Alginate	2.2	89.0	4.96	10.4	123.5	17.578
Al_2_O_3_	3.8	9.0	0.91	4.6	43.5	4.41
Al_2_O_3_ + Sodium alginate	3.4	28.0	2.84	12.9	103.5	14.53
Latex	2.4	34.0	2.43	4.5	98.6	5.92
Latex + Sodium alginate	4.6	94.0	6.48	10.8	148.6	6.77
SiO_2_	2.9	24.0	3.46	4.6	61.7	5.27
SiO_2_ + Sodium alginate	2.9	106.2	6.79	9.2	111.9	4.21

## Data Availability

The raw data supporting the conclusions of this article will be made available by the authors on request.

## Abbreviations

The following abbreviations are used in this manuscript:

NF	Nanofiltration
RO	Reverse Osmosis
CP	Concentration Polarization
CECP	Cake-Enhanced Concentration Polarization
SD	Solution-Diffusion
